# Graphene photodetectors integrated with silicon and perovskite quantum dots

**DOI:** 10.1038/s41378-024-00722-4

**Published:** 2024-06-20

**Authors:** Kashif Abbas, Peirui Ji, Naveed Ullah, Shareen Shafique, Ze Zhang, Muhammad Faizan Ameer, Shenghan Qin, Shuming Yang

**Affiliations:** 1 State Key Laboratory for Manufacturing Systems Engineering, Xi’an Jiaotong University, Xi’an, 710049 China; 2 Department of Microelectronic Science and Engineering, Laboratory of Clean Energy Storage and Conversion, School of Physical Science and Technology, Ningbo Collaborative Innovation Center of Nonlinear Calamity System of Ocean and Atmosphere, Ningbo University, Ningbo, 315211 China

**Keywords:** NEMS, Nanoparticles

## Abstract

Photodetectors (PDs) play a crucial role in imaging, sensing, communication systems, etc. Graphene (Gr), a leading two-dimensional material, has demonstrated significant potential for photodetection in recent years. However, its relatively weak interaction with light poses challenges for practical applications. The integration of silicon (Si) and perovskite quantum dots (PQDs) has opened new avenues for Gr in the realm of next-generation optoelectronics. This review provides a comprehensive investigation of Gr/Si Schottky junction PDs and Gr/PQD hybrid PDs as well as their heterostructures. The operating principles, design, fabrication, optimization strategies, and typical applications of these devices are studied and summarized. Through these discussions, we aim to illuminate the current challenges and offer insights into future directions in this rapidly evolving field.

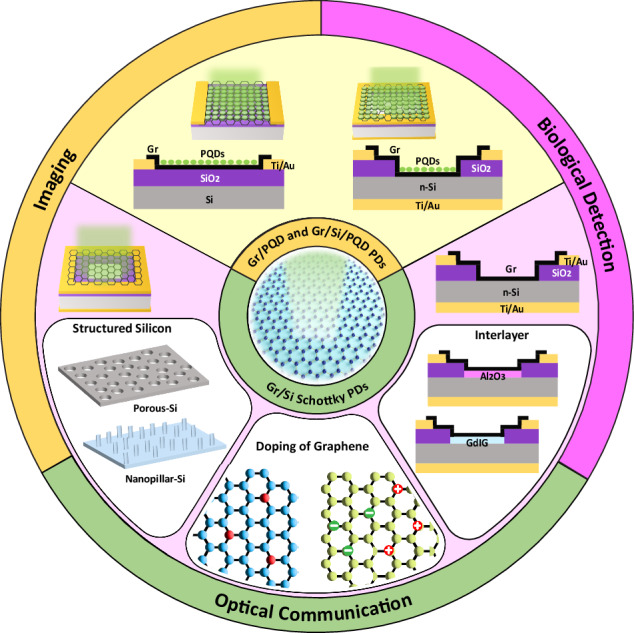

## Introduction

Photodetectors (PDs) are fundamental optoelectronic devices that convert optical signals into electrical signals. They play an important role in several sectors, including industrial production, optical communications, and biomedical imaging. Conventional bulk semiconductors, such as silicon (Si), indium gallium arsenide, and mercury cadmium telluride, have been good candidates for PDs in recent decades^1–3^. However, their performance is limited by their inherent bandgap, doping level, and thermal noise. Graphene (Gr) is a single layer of carbon atoms arranged in a honeycomb lattice. It possesses a tunable bandgap, broad absorption spectrum, and ultrafast carrier dynamics, making it a promising contender for optoelectronics^4,5^. However, the light absorbance of pristine Gr is only 2.3% in the visible and near-infrared bands^6^, severely limiting the photoresponsivity of Gr-based PDs. To enhance light-matter interactions, significant efforts have been directed toward integrating Gr with other photosensitive materials^7–11^ or embedding it into nanophotonic structures^12–19^. Among these approaches, integration with Si^20^ and perovskite quantum dots (PQDs)^21,22^ has garnered widespread attention in recent years.

Benefiting from their two-dimensional (2D) planar nature, heterojunctions can be easily built when Gr is stacked onto a Si surface^23–26^. The different working functions of Gr and Si create a Schottky barrier at the interface, which enables rectification and controls the carrier flow. The Gr/Si Schottky junction has been developed in high-performance PDs, where Gr serves not only as the junction’s active layer but also as a transparent electrode. In this configuration, light is predominantly absorbed within the thick bulk Si layer, resulting in a high photoresponse that covers ultraviolet to near-infrared wavelengths.

Perovskites, known for their distinctive ABX_3_ crystal structure, have received significant attention due to their exceptional optoelectronic properties^27,28^. Compared with bulk perovskites, PQDs have advantages in terms of property adjustment through quantum confinement^29,30^. PQDs have been demonstrated to be excellent optical materials with high absorption coefficients and long carrier diffusion lengths. Due to their solution processability, Gr/PQDs hybrid PDs can be fabricated using a simple spin-coating method^31,32^. In this architecture, PQDs typically serve as light absorbers, while Gr acts as a carrier transport pathway. The remarkable absorption of the PQDs and the fast charge transport facilitated by Gr contributed to the formation of a high-performance photodetector.

**Fig. 1:**
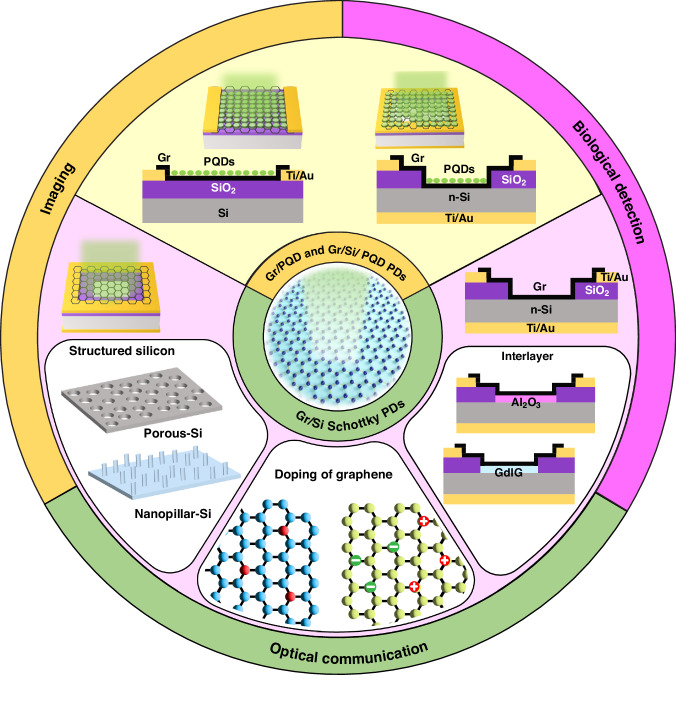
Outline of this review. Schematic illustration of Gr/Si Schottky PDs, Gr/PQD PDs, and Gr/Si/PQD hybrid PDs, as well as their optimization methods and applications

This review offers a comprehensive analysis of Gr PDs integrated with Si and/or PQDs. We start by discussing the fundamental mechanisms of photodetection and the key performance metrics. Subsequently, we delve into a systematic investigation of the design, fabrication, operating principles, and optimization methods of Gr/Si and Gr/PQD PDs based on recent advancements. Finally, we analyze the representative applications of the PDs under review and address the current challenges and future perspectives in this field. Figure 1 depicts the navigation path throughout the review. This comprehensive study aimed to enhance the understanding of the landscape of hybrid Gr-based PDs, providing insights into their potential applications.

## Photodetection mechanisms and figures-of-merit

**Fig. 2:**
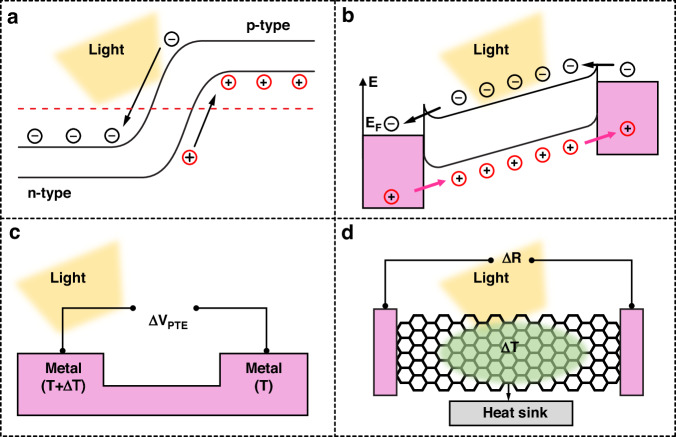
Typical photodetection mechanisms. **a** Photovoltaic effect, **b** photoconductive effect, **c** photothermoelectric effect, and **d** bolometric effect

The fundamental operating principle of Gr PDs involves the separation of electron-hole (e–h) pairs generated by the absorption of light. Various photodetection mechanisms have been developed and employed, including the photovoltaic effect, photoconductive effect, photo thermoelectric effect, and bolometric effect^33,34^. In the photovoltaic effect (Fig. 2a), charges are generated upon absorbing photons with higher energy than the bandgap of the material. These charges are then separated by the built-in electric field at the p-n junction^35^. The photoconductive effect (Fig. 2b) occurs when high-energy photons create additional free carriers, increasing the electrical conductivity of the semiconductor material^36^. These carriers are further separated by applied bias voltages, leading to a net photocurrent. The photothermoelectric effect (Fig. 2c) involves the local illumination of light^37^, which uniformly increases the material temperature, $\triangle$T, due to the Seebeck effect. The resulting temperature gradient produces a voltage difference, ${\triangle V}_{{PTE}}$, that drives a detectable current. When light illumination is homogeneous and permits uniform heating, the photodetector can operate via the bolometric effect (Fig. 2d). Here, an increase in the homogenization temperature alters the resistivity of the material, $\triangle$R, consequently modifying the current magnitude under an external bias^38^. The devices reviewed in this paper are mainly based on the photovoltaic effect and photoconductive effect.

The metrics for PDs mainly include the responsivity (R), on/off ratio (I_ON/OFF_), detectivity (D), noise equivalent power (NEP), dark current, response speed, and quantum efficiency^39^. Responsivity measures the output electrical signal per unit input optical power, indicating the efficiency of converting light to an electrical signal. The on/off ratio compares the output when illuminated to the output in the dark, serving as a measure of the device’s ability to distinguish between light and no light conditions. Detectivity quantifies the ability to sense weak signals, defined as the signal-to-noise ratio of the device for a given bandwidth and area at a specific wavelength (λ). NEP represents the noise level, expressed as the power of the incident light that generates a signal equal to the noise level, with lower values indicating less noise. The dark current refers to the current that flows through the photodetector when no light is present, with lower dark currents indicating less noise. The response speed is divided into rise time (RT, the time needed for the output signal to increase from 10% to 90% of its maximum value upon receiving light) and fall time (FT, the time needed for the output to decrease from 90% to 10% of its maximum value after the light is removed). Quantum efficiency is categorized into internal quantum efficiency (IQE) and external quantum efficiency (EQE), where IQE measures the efficiency of converting absorbed photons into charge carriers within the device, and EQE considers both the absorption of photons and their conversion into charge carriers, reflecting the overall efficiency of the device in converting incident photons into an electrical signal.

## Gr/Si Schottky photodetector

The mechanical cleavage of graphite into single-atom layers led to the discovery of Gr, the first 2D material^4^. Gr exhibits a zero band gap due to the linear dispersion around the Dirac point, resulting in semimetallic properties^5^. Because of this unique structure, Gr can absorb broadband wavelengths from the ultraviolet (UV) to the infrared (IR) range. However, a single sheet of Gr has a measured thickness of ~0.34 nm, which limits its absorbance to only 2.3% for normal incident light^6^. The simple transfer process of the Gr membrane onto three-dimensional Si enables the formation of a Gr/Si heterostructure between the two materials at a shallow depth. Three-dimensional Si has gained significant popularity as a junction material for photodetection due to its high absorbance and well-established fabrication technique. In the Gr/Si hybrid structure^23,24,40,41^, the incident light stimulates the generation of carriers, most of which are generated in Si, and Gr serves as a carrier collector and transfer media. This approach takes advantage of the fast carrier dynamics of Gr and the high absorbance of Si to revolutionize the fields of micro/nanoelectronic devices.

**Fig. 3:**
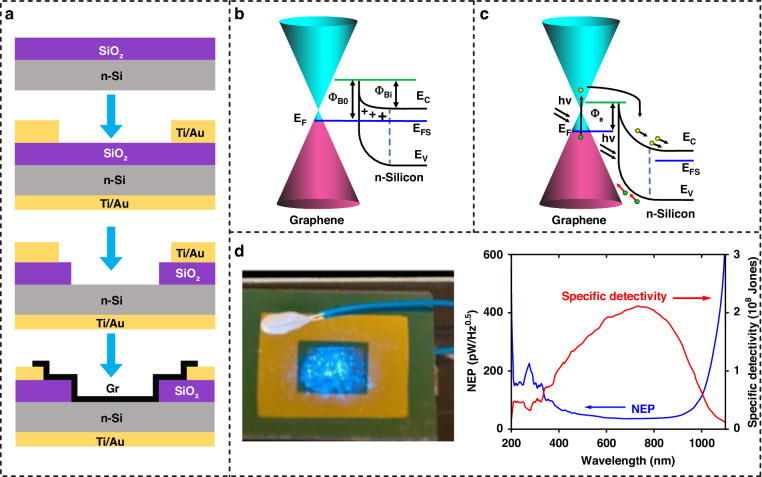
Gr/Si Schottky junction PDs. **a** Fabrication process, **b** band diagram in the dark and **c** under illumination, and **d** a representative work based on the Gr/Si Schottky junction and its wavelength-dependent NEP and specific detectivity. Reproduced with permission from ref. ^24^

The typical Gr/Si PD structure and its fabrication process are shown in Fig. 3a. This process begins with the patterning and evaporating of the bottom and top electrodes on a Si/SiO_2_ wafer. Thin layers of titanium (Ti) and gold (Au) are employed to ensure ohmic contact. Then, a square window is defined and etched in the SiO_2_ layer to expose the n-type Si (n-Si) surface. Subsequently, the CVD-grown Gr membrane is wet-transferred onto the Si window and the top electrodes with the assistance of polymethyl methacrylate (PMMA), which naturally forms a Gr/Si junction. Gr is transferred in the last step so that it is not contaminated by the forward fabrication process. Figure 3b shows the band structures of the Gr/Si Schottky junction in the dark. The Fermi levels (E_F_) of Gr and Si align due to thermal equilibrium when they are in contact. The built-in electric field creates a Schottky barrier height (Φ_B_) at the interface. Figure 3c displays the band structure when the junction is illuminated. Photons with energies lower than the Si bandgap can be absorbed, producing photogenerated carriers that are further separated by the built-in electric field. A photocurrent is generated when holes are injected into Gr, while electrons move into Si. By applying a bias voltage, one can modulate the Schottky barrier height of the junction^26^, thus modulating the photocurrent signals.

Gr/Si Schottky junctions have demonstrated high efficiency in photodetection applications in the past few years^25,42^. The detailed performances of recent works involving the basic Gr/Si Schottky architecture are summarized at the beginning of Table 1. For example, An et al.^24^ comprehensively studied the optoelectronic performance of a Gr/Si Schottky PD, as shown in Fig. 3d. They revealed that the device could operate in both photovoltage and photocurrent modes. It exhibited a spectral response from the UV to near-IR regime with a high photoresponse. Since Gr primarily serves as a transparent electrode and a high-speed carrier transport channel here, light absorption is mainly governed by Si; thus, the spectral range is limited by the Si bandgap. For weak incident signals, the voltage responsivity reached 107 V/W but rapidly decreased with increasing incident power. By adjusting the E_F_ through bias voltage, the detector exhibited adjustable responsivity, reaching a maximum of 0.435 A/W at 850 nm. The response speed of the detector was limited by the carrier mobility of Si but still reached milliseconds.

**Table 1:** Performance of the Gr/Si Schottky PDs and the optimization with doped Gr, structured Si, and interlayers

Ref	Materials	R (A/W)	D (Jones)	λ (nm)	Other parameters
Basic Gr/Si					
^23^	Gr/Si		1.6 $\times$ 10^11^–1.9$\times$10^9^	1500–4000	
^24^	Gr/Si	0.435		400–900	
^40^	Gr/Si	0.3	3.37$\,\times \,$10^11^	532	EQE = 90%
^41^	Gr/Si	0.2	1.6 $\times$ 10^13^		
Doped Gr					
^43^	MoO_3_-doped Gr/Si		5.4$\,\times$ 10^12^	600–800	EQE ≈ 80%
^44^	Boron-doped Gr/Si			365	I_ON/OFF_ = 1.5 $\times$ 10^4^
^45^	PEI-doped Gr/Si		5.9$\,\times \,$10^10^	850	
^46^	p-doped Gr/Si	45000		532	
^47^	Electrostatically-doped Gr/Si	1.1$\,\times \,$10^4^		635	
^48^	Electrostatically-doped Gr/Si	10^7^	1.46 $\times$ 10^13^	532	
^49^	Electrostatically-doped Gr/Si	3	3.5 $\times$ 10^12^		
Structured Si					
^50^	Gr/PSi	0.35		950	
^51^	Gr/Si grating	240	3.4 $\times \,$10^12^	1550	
^52^	Gr/Si NWs	1.5	2.52$\,\times \,$10^14^		I_ON/OFF_ > 10^6^, RT/FT = 73/96 μs
^53^	Gr/Si pillar	88			
^54^	Gr/Si nano-needle				EQE > 130%
^55^	Gr/Si photonic crystal cavity			1550	Absorptance = 85%
Interlayer					
^56^	Gr/SiO_2_/Si	0.73	5.77 $\times$ 10^13^	890	
^57^	Gr/GO/Si	0.65	2.73 $\times$10^5^	633	
^58^	Gr/Al_2_O_3_/Si	0.75	3.1 $\times$ 10^12^	658	
^59^	Gr/MoS_2_/Si	3 $\times$ 10^4^		635	RT/FT = 17 ns/1.1 μs
^60^	Gr/AlN/Si	3.96	1.13$\,\times$ 10^8^	850	
^61^	Gr/GdIG/Si		1.35 $\times \,$10^13^	633	I_ON/OFF_ = 8.2 × 10^6^

**Fig. 4:**
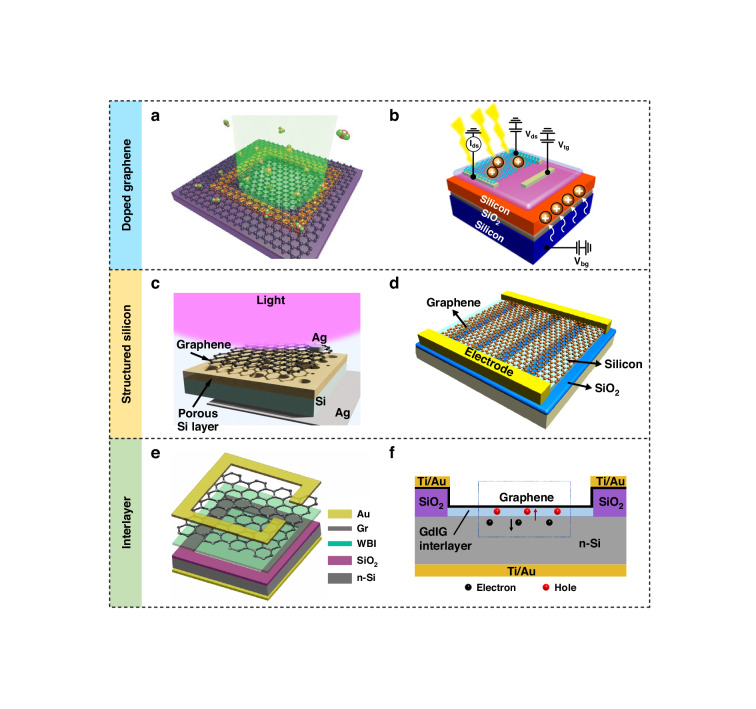
Optimization techniques for the Gr/Si Schottky PDs. **a** Device schematic of surface-transfer-doped Gr/Si Schottky PD. Reproduced with permission from ref. ^43^. **b** Device structure of an ionic-polymer-gate-doped Gr/Si PD. Reproduced with permission from ref. ^47^. **c** Schematic diagram of Gr/porous-Si PD. Reproduced with permission from ref. ^50^. **d** Schematic diagram of a hybrid PD composed of Si grating and monolayer Gr. Reproduced with permission from ref. ^51^. **e** Demonstration of a Gr/Si Schottky PD with a wide bandgap insulating (WBI) layer. Reproduced with permission from ref. ^60^. **f** Schematic of a Gr/Si Schottky PD with an interlayer of gadolinium iron garnet (GdIG) film. Reproduced with permission from ref. ^61^

Due to the nature of the junction, the performance of Gr/Si Schottky PDs largely relies on the cleanness of the interface, the built-in electric field, and the Schottky barrier height. Although Gr/Si Schottky PDs have shown high-performance broadband photodetection, their specific detectivity is limited by the large dark current. Additionally, due to the lack of a photocurrent gain mechanism, the responsivity of these materials is limited to hundreds of mA/W. To address these challenges, efforts have been made toward the doping of Gr^43–49^, the structurization of planar Si^50–55^, and interface engineering^56–62^. We summarize recent works involving optimization methods in Table 1 and interpret the representatives that are presented in Fig. 4.

### Doped-Gr/Si Schottky PD

Doping is an efficient method to modulate the carrier density and band structure of Gr-based PDs, significantly enhancing their photoresponse properties. The introduction of chemical dopants and electrostatic charging are the typical methods involved in a PD architecture. Figure 4a shows the device structure of a Gr/Si photodetector constructed from surface-transfer-doped Gr and planar Si^43^. In this work, a MoO_3_ thin film is adopted to induce hole doping on Gr, which further increases the Schottky barrier height at the interface and reduces the series resistance of the device. The external quantum efficiency of the doped device is enhanced up to ≈80%. In addition, Liu et al. fabricated a hybrid Gr/Si PD by boron-doped Gr^44^. Boron-doped Gr presents p-type characteristics with a larger Schottky barrier height than pristine Gr when in contact with Si. The device shows an on/off ratio of 1.5$\times$10^4^ in self-power mode. Solution-based dopants are also widely used to introduce doping to Gr via chemical reactions or interactions. Yoo et al. demonstrated the efficient doping of Gr with polyethyleneimine (PEI) solution, which helps to increase the barrier height up to 0.26 eV and reduce the dark current by four orders of magnitude^45^. Figure 4b shows the electrostatic doping of a Gr/silicon-on-insulator PD by an ionic-polymer gate^47^. Ionic polymers are newly emergent dielectric media that help to improve the switching speed of Gr-based PDs. The device gains an enhanced responsivity (1.1 $\times$ 10^4^ A/W at 635 nm and 15 A/W at 1550 nm) and response speed by modulating the carrier distribution and transport at the junction. Doping techniques have also been applied to the optimization of other Gr-based PDs.

### Gr/Structured-Si Schottky PD

Structured Si increases the ratio of surface area to volume and generates localized enhancement effects^63,64^. The application of nanostructured Si in a Gr/Si Schottky junction holds immense potential for enhancing the photodetection performance in terms of high light absorbance and high optical gain. Additionally, the flexibility in dimensions helps to tailor the band structure and corresponding response spectrum. Typical types of nanostructured Si include porous Si (PSi), Si pillars, Si nanowires, and gratings. Figure 4c shows an example of a Gr/PSi PD^50^. The spectral response shows sensitivity at near-UV wavelengths, which can be understood by the enlarged energy band gap of PSi. The response speed of the device is ∼10 times faster than that of the Gr/planar Si. Figure 4d shows an optimization method based on potential fluctuation engineering enabled by Si grating^51^. The photogating effect and the built-in potential are largely enhanced, facilitating efficient separation and harvesting of the photogenerated carriers. The device reaches a specific detectivity of 3.4 $\times \,$10^12^ Jones and a responsivity of 240 A/W at 1550 nm, and it has dominant advantages over devices without nanostructured Si.

### Insertion of interlayers

The quality of the junction interface between Gr and Si is of vital importance for the behavior of a Gr/Si PD. Incorporating interlayers into Gr/Si Schottky PDs plays a crucial role in manipulating the band alignment and charge carrier dynamics and in optimizing the interface properties, thus emerging as a compelling avenue to enhance the photodetection performance. For the first time, Li et al. used a thermally grown native SiO_2_ layer in a Gr/Si Schottky PD, which improved the specific detectivity due to the suppression of the dark current. The Gr/SiO_2_/Si hybrid device shows a detectivity of 5.77$\times$10^13^ Jones and a light/dark ratio of 10^7^ at 890 nm. However, the disadvantage of the native oxide layer is the continuous increase in thickness, which further blocks the tunneling of the generated e-h pair^56^. In addition, researchers have identified several fascinating interlayer materials, such as graphene oxide (GO)^65^, aluminum oxide (Al_2_O_3_), molybdenum disulfide (MoS_2_), aluminum nitrate (AlN), and gadolinium iron garnet (GdIG). Wang et al. demonstrated a dark current suppression of >10 times after the insertion of the GO interlayer, which also helped to improve the on/off ratio to 2.73$\times$10^5^ ^57^. Xu et al. fabricated a Gr/Al_2_O_3_/Si Schottky junction PD with a 13.3 nm thick Al_2_O_3_ interlayer, which exhibited a maximum photoresponsivity of 0.75 A/W, a specific detectivity of 3.1$\times$10^12^ at 658 nm and an on/off ratio of almost 4.8$\times$10^3^ ^58^. Tao et al. developed a Gr/MoS_2_/Si hybrid Schottky junction by employing the atomically thin 2D semiconductor MoS_2_^59^. The responsivity of the Gr/MoS_2_/Si device was improved to 3$\times$10^4^ A/W at 635 nm. Another advantage of the MoS_2_ interlayer is the increase in the response speed by ~ 3 orders of magnitude compared to that of the reference Gr/Si architecture. Yin et al. used the band gap engineering technique to demonstrate a high-performance Gr/Si Schottky PD with a wide bandgap insulating (WBI) layer of AlN, as shown in Fig. 4e. The 15.3 nm thick AlN interlayer increases the photogain and reduces the dark current owing to the enhanced impact ionization. The responsivity reached 3.96 A/W at a -10 V reverse bias under 850 nm light illumination, which is superior to that of control devices with Al_2_O_3_ and SiO_2_ interlayers^60^. GdIG is a high-k material with good temperature and chemical stability. As illustrated in Fig. 4f, Ji et al. used a sputtered thin layer (~2 nm) of GdIG as an insulating layer in a Gr/Si Schottky PD^61^. This new structure exhibits a significant decrease in the dark current, 54 times lower than that of the conventional Gr/Si PD, which enables high sensitivity for weak-light detection. The device has an operation speed of 0.15 ms, a stable response for multiple working cycles, and long-term environmental stability. The dielectric constant, thickness, and stability of the interlayer materials are crucial aspects to consider when constructing such a sandwiched Gr/Si Schottky photodetector.

Many advantages of the optimization methods and their respective applications in PDs have been discussed in the previous subsections. However, these techniques also have several limitations. For example, doping may introduce defects or impurities into the Gr lattice, affecting its electronic properties and PD performance. Moreover, precise control over doping levels can be challenging. The limitations of structured Si in PDs are the complexity of the device structure and the increased manufacturing cost. Additionally, structural modifications may introduce defects or nonuniformities that could degrade device performance. Furthermore, achieving precise control of the interface properties requires sophisticated fabrication or growth techniques, and the interface engineering approaches are sensitive to environmental factors, such as temperature and humidity.

## Gr/PQD hybrid photodetector

Perovskite originates from a mineral ore characterized by the chemical formula CaTiO_3_. In recent years, metal halide perovskites have emerged as a revolutionary class of materials that reshape the landscape of optoelectronic devices^66^. They share a crystal structure similar to that of CaTiO_3_, with a standard formula ABX_3_, where ‘A’ is a monovalent cation, ‘B’ is a divalent metal ion, and ‘X’ is a halide anion (Cl^−^, Br^−^, or I^−^^67,68^). QDs are nanoscale semiconductor particles that exhibit quantum mechanical properties that distinguish them from bulk materials. PQDs offer an advantage over bulk perovskites due to quantum confinement^69,70^. They hold great potential for applications in optoelectronics due to their tunable bandgap, high quantum yield, and solution processability.

**Fig. 5:**
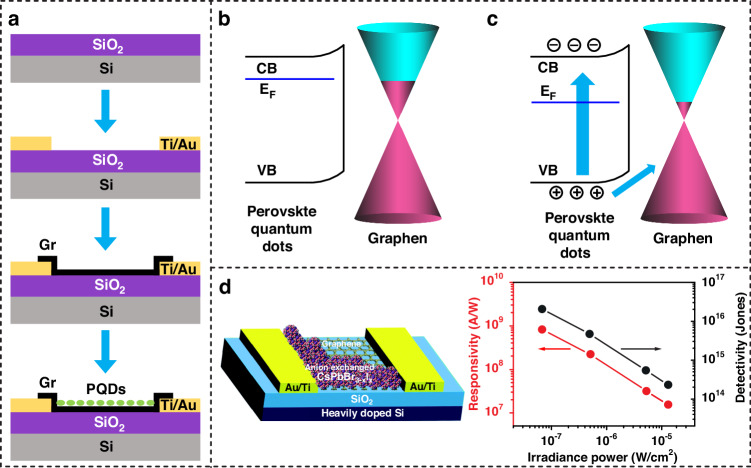
Gr/PQD PDs. **a** Fabrication process, **b** band diagram in the dark and **c** under illumination. **d** A representative work based on the Gr/PQD structure and its power-dependent responsivity and detectivity. Reproduced with permission from ref. ^77^

PQDs can be coated over a Gr film to form a Gr/PQD hybrid architecture in which PQDs normally serve as light absorbers, while Gr transports the carriers to form a current flow^71^. With proper band alignment, the PQDs modulate and enhance the built-in electric field at the junction interface, facilitating the efficient separation of photogenerated carriers. Figure 5a presents the fabrication process of this hybrid device. The Gr is transferred onto a Si/SiO_2_ substrate with prefabricated metal layers that serve as source-drain electrodes. PQDs are then integrated vertically with Gr using a spin-coating process. Figure 5b, c depict the band structure of the Gr/PQD junction in the dark and under illumination conditions. Initially, in the separated state, the conduction band (CB) of the PQDs is marginally larger than the E_F_ of Gr, facilitating favorable conditions for electron transfer from the PQDs to Gr. The valence band (VB) of the PQDs is located below the E_F_ of Gr, enabling hole transfer from Gr to the PQDs. Subsequently, the bands of the Gr and PQDs align with the E_F_ of the heterojunction. Charge transfer can occur with an external bias between Gr and the PQDs. Upon illumination, incident photons elevate electrons within the PQDs to the CB, instigating the generation of electron-hole pairs. The movement of e-h pairs, intricately regulated by energy level alignments and interfacial interactions, fundamentally governs the electrical behavior and performance of the device. Multiple studies have demonstrated that the combination of Gr and PQDs results in high-performance PDs^21,71–81^, as summarized in Table 2. For example, Fig. 5d shows a diagram of the Gr/CsPbBr_3_-xIx colloidal quantum dot junction, in which the built-in field gives rise to a barrier height, facilitating the generation of photogenerated current^77^. The device shows considerable performance in terms of a responsivity of 8.2 $\times$10^8^ A/W and a D* of 2.4$\times$10^16^ Jones under an optical power of 0.07 µWcm^−2^ at 405 nm.

**Table 2:** Performance of the Gr/PQD-based hybrid PDs

Ref	Materials	R (A/W)	D (Jones)	λ (nm)	Other parameters
^21^	Gr/FAPbBr_3_	1.15$\,\times \,$10^5^		520	EQE = 3.42$\,\times \,$10^7^%
^71^	Gr/FAPbI_3_	2.17$\,\times$ 10^7^	5.64$\,\times$ 10^15^	1550	RT/FT = 40/46 µs
^72^	Gr/FAPbI_3_	8.03	1.89 $\times \,$10^10^	365	
^73^	Gr/MAPbI_3_	10^7^		633	
^74^	Gr/CsPbBr_3_	3.4	7.5 $\times$ 10^8^	405	EQE = 10^3^%
^75^	Gr/CsPbBr_3_	2 $\times$ 10^4^	8.6 $\times$ 10^10^		EQE = 6 $\times$ 10^6^%, RT/FT = 3.1/24.2 s
^76^	Gr/CsPbBr_3_	22.8	1.89 $\times$ 10^10^	266	
^77^	Gr/CsPbBr_3-x_I_x_	8.2$\,\times$ 10^8^	2.4$\,\times$ 10^16^	405	
^78^	Gr/CH_3_NH_3_PbBr_3_	3$\,\times$ 10^9^	8.7 $\times$ 10^15^		RT = 20 µs, EQE ≈ 1.2$\,\times$ 10^10^%
^79^	Gr/Au/MAPbBr_3_	2.7 $\times$ 10^5^	4.9 $\times$ 10^13^	432	EQE = 7.9 $\times$ 10^7^%
^80^	GNR/CsPbBr_3_/IGZO	1 $\times$ 10^5^	7.5$\,\times \,$10^14^		EQE = 5 $\times$ 10^5^%, I_ON/OFF_ > 10^3^
^81^	Gr/Si/CsPbBr_3_	0.73	10^12^	400	

**Fig. 6:**
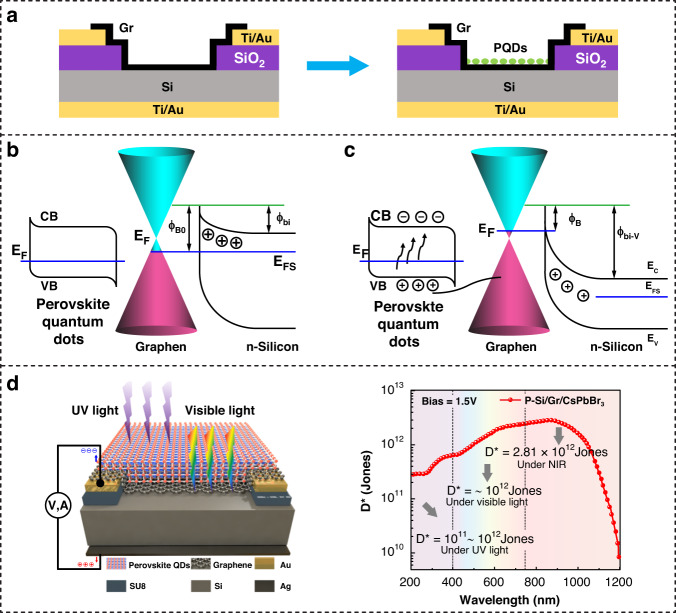
Hybrid PDs based on a Gr/Si/PQD sandwiched structure. **a** Fabrication process based on a predefined Gr/Si Schottky junction. **b** Energy band diagram of the Gr/Si/PQD PD in the dark and **c** under illumination. **d** A representative work based on the Gr/Si/PQD structure and its wavelength-dependent detectivity. Reproduced with permission from ref. ^81^

PQDs serve a crucial function in enhancing light absorption and charge carrier generation due to their tunable bandgap, high absorption coefficient, and efficient charge transfer properties. The distinct qualities of PQDs coupled with the flexibility of device construction via solution processing enable the creation of complex optoelectronic devices. The integration of PQDs into a Gr/Si Schottky junction is another newly emerging hybrid architecture that combines the advantages of tunable absorption and long carrier lifetime of PQDs and the fast carrier separation of the Gr/Si Schottky junction. As shown in Fig. 6a, an additional process of spin-coating on the fabricated Gr/Si Schottky junction gives rise to a hybrid sandwiched Gr/Si/PQD architecture. When integrated into the Gr/Si junction, PQDs absorb incident photons across a wide spectral range, and the generated electron-hole pairs quickly transfer to the Gr layer due to high conductivity. This mechanism facilitates improved photodetection capabilities, leading to enhanced detectivity and sensitivity in the Gr/Si/PQD PD. Figure 6b shows the band diagrams of the Gr/Si/PQD PD in the absence of light. A Schottky junction exists at the interface of the Gr layer and the *n*-type Si. The built-in potential barrier (**Φ*bi***) and the barrier height (**Φ**_*B*o_) at the interface are produced by the different work functions of Gr and Si. The existence of the PQDs improves the potential of the heterojunction by the electrical coupling mechanism between perovskite QDs and Gr^81^. Figure 6c shows the results under light illumination. Incident photons are absorbed mainly by the PQDs and Si, resulting in the generation of e-h pairs. The E_F_ of Gr shifts as photogenerated carriers are injected. The enhanced built-in potential promotes the separation of free carriers. Current reports on this new type of device structure remain scarce. Tan et al.^81^ integrated high-quality perovskite colloidal quantum dots (CsPbBr_3_ QDs) with a Gr/Si heterojunction to broaden the spectral response by effectively utilizing the strong UV absorption of PQDs. The device structure is shown in Fig. 6d. After fabricating the Gr/Si junction, the PQDs films are cast on the Gr/Si window. As shown in the spectral response curve, the enhanced Si/Gr PD displays a superior detectivity of nearly 10^12^ Jones and a high responsivity of 0.73 A/W in the deep-UV region.

While the hybrid Gr/Si/PQD PDs exhibited promising performance improvements, the fabrication process of these structures is relatively complex. Furthermore, the incompatibility of materials at the interface between Gr, Si, and quantum dots can cause defects and inhomogeneities, thereby degrading device performance. Additionally, hybrid structures may exhibit different material properties, such as different thermal expansion coefficients or susceptibilities to environmental factors, leading to potential reliability problems over time. Therefore, the practical applicability of this device structure requires further study.

## Applications of the PDs

### Imaging

**Fig. 7:**
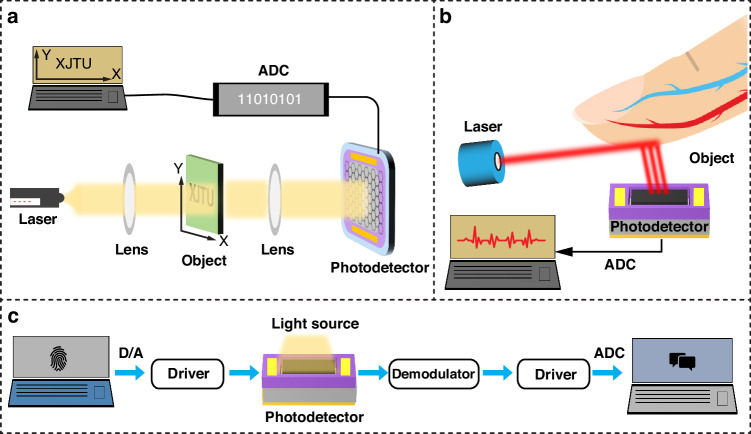
Schematic diagram of the applications of the Gr hybrid PDs. **a** Imaging system, **b** biological detection, and **c** optical communication system

Imaging is one of the most important applications for PDs. The principle of modern digital image sensors is similar to the process of human eye imaging, which uses a combination of array mode receiving units to convert external light signals into electrical signals that are then processed and restored to image information. Figure 7a depicts a standard photographic configuration based on Gr hybrid PDs. Light is emitted from a laser source and ultimately converges onto a photodetector array through a color lens, imaging template, and focusing lens. By moving the object template through the x-y stage, different positions of light and dark information can be obtained. The analog signal converted by the image sensor is amplified and converted by the analog-to-digital module and sent to the computer. It is then restored to an image through grayscale and color processing and finally displays the image of the objective. Many developments in Gr PD-based imaging sensors have relied on similar principles^82,83^.

### Biological detection

Due to the advantages of miniaturized real-time feedback and noninvasiveness, Gr-based hybrid PDs play an important role in biological detection and intelligent health care. The optical signals emitted or reflected by human tissues carry physiological information about the organism, which can be used to noninvasively monitor health conditions by capturing and converting spectra into electrical signals. In the field of intelligent medical optical detection, PDs can non-destructively monitor the vital signs of living organisms using the photo-capacitive pulse wave tracing method, providing convenient and fast medical services. These capabilities allow Gr PDs to be used in skin cancer prevention, blood spot age estimation, protein detection, infrared imaging, and blood analysis. Figure 7b shows a method for detecting heart rate and blood oxygen saturation. A laser source illuminates specific wavelengths of light on human tissues, the transmitted light signals are collected through PDs, and the light signals are analyzed to obtain personal physiological information. Specifically, the biocompatibility and surface sensitivity of Gr make it ideal for biological sensing applications, including measurements of heart pulse rate, blood oxygen saturation, blood pressure, blood volume, and other personal health indicators^84^.

### Optical communication

PDs are important modules in optical communication systems. They are responsible for detecting long-distance weak light signals with a fast response speed. Figure 7c shows a schematic diagram of the working principle of optical communication, which consists of a modulation module, a receiving detector, a demodulation module, and a receiving system. The information in the transmission system is converted into analog signals through digital-analog conversion. The transmission light is modulated by the modulator and detected and converted into an electrical signal by a photodetector. The demodulation module converts electrical signals into digital signals and transmits them to the receiving system, thus completing the process of optical communication. The reaction time of these devices directly determines the system bandwidth. The rapid carrier dynamics and high carrier mobility of Gr make it suitable for high-speed photodetection; consequently, it is an ideal material for optical communication systems. Hybrid structures can further enhance the responsivity and bandwidth, enabling efficient data transmission^85^.

## Perspectives and challenges

The integration of Gr, Si, and PQDs in PDs represents a cutting-edge approach that harnesses the unique advantages of different materials and heterojunctions to achieve exceptional performance. Hybrid PDs offer promising prospects for next-generation devices in terms of flexible and wearable electronics, the sensitive detection of broadband weak signals^86^, and low-cost production. Wearable devices require portable and flexible PDs. Gr has exceptional mechanical properties, enabling the development of bendable and durable PDs^87,88^. When combined with different semiconductors, Gr hybrid PDs have great potential for multifunctional properties^89^. By combining the high electron mobility and excellent conductivity of Gr with the high light absorption coefficient and tunable bandgap of perovskite materials, hybrid PDs offer significant advantages for detecting weak light signals in applications such as long-distance imaging^90^. The cost of Gr-based detectors can vary widely depending on several factors, but since Gr itself is a carbon-based material, it can be synthesized from a variety of natural materials, such as graphite or even biomass, offering possibilities for low-cost production costs.

However, challenges persist in meeting the requirements for practical applications in terms of environmental friendliness, integration, complementary metal‒oxide‒semiconductor (CMOS) compatibility^91^, and long-term stability^92^. Despite their excellent performance, the toxic heavy metals (especially lead and Pb) in PQDs pose significant environmental and health concerns^93^. These concerns include the potential for hazardous elements to leak into the environment and risks associated with the fabrication process. To address this issue, researchers are exploring nonlead PQDs, such as tin (Sn) or other less toxic metals^94,95^, which are eco-friendly. The practical application performance highly relies on the quality of these low-dimensional materials and their interfaces. The hybrid device structures reviewed above are generally assembled by transfer, coating, and lithography processes. Due to its low-dimensional nature, Gr is susceptible to wrinkling, contamination, and damage. High-quality integration requires effective growth and assembly while maintaining the intrinsic material properties. Recent advancements have significantly improved growth techniques for producing large-scale 2D materials^96,97^. However, the assembly of different materials limits their CMOS compatibility. One of the key challenges lies in the seamless integration of Gr transfer technology into the back-end-of-line process in CMOS technology^98^. One potential solution involves the direct growth of heterostructures on top of each other^70^. The stability of Gr-based hybrid PDs over an extended period is of vital importance for successful implementation in commercial applications. In general, Gr exhibits good long-term stability due to the strong sp2 hybridization of carbon atoms, which results in the formation of a dense honeycomb lattice^99^. However, PQDs are sensitive to environmental factors, such as moisture, oxygen, and temperature, stemming from the ionic nature of perovskite materials; specifically, the crystal structure can be easily disrupted by external conditions^100^. Proper surface passivation and encapsulation are needed to ensure that hybrid PDs are stable long term.

## Conclusion

In conclusion, this article presents a comprehensive overview of Gr PDs integrated with Si and PQDs. The material properties and photodetection mechanism are discussed and analyzed. The operating principles, design, fabrication, optimization methods, and typical applications of Gr/Si Schottky junction PDs and Gr/PQD hybrid PDs are studied and summarized based on recent advancements. The hybrid device structures show advanced performance in terms of high detectivity and responsivity within a broadband spectrum and have great potential for flexible electronics and low-cost production. However, challenges, such as mass production and long-term stability, persist. This review offers significant perspectives on the progress and innovation in Gr-based hybrid devices.
